# *Fasciola hepatica* Kunitz Type Molecule Decreases Dendritic Cell Activation and Their Ability to Induce Inflammatory Responses

**DOI:** 10.1371/journal.pone.0114505

**Published:** 2014-12-08

**Authors:** Cristian R. Falcón, Diana Masih, Gerardo Gatti, María Cecilia Sanchez, Claudia C. Motrán, Laura Cervi

**Affiliations:** 1 Department of Biological Chemistry, Faculty of Chemical Sciences, National University of Cordoba, CIQUIBIC-CONICET, Córdoba, Argentina; 2 Department of Clinical Biochemistry, Faculty of Chemical Sciences, National University of Cordoba, CIBICI-CONICET, Córdoba, Argentina; 3 Foundation for the advancement of Medicine, Córdoba, Argentina; Institut National de la Santé et de la Recherche Médicale U 872, France

## Abstract

The complete repertoire of proteins with immunomodulatory activity in *Fasciola hepatica* (Fh) has not yet been fully described. Here, we demonstrated that Fh total extract (TE) reduced LPS-induced DC maturation, and the DC ability to induce allogeneic responses. After TE fractionating, a fraction lower than 10 kDa (F<10 kDa) was able to maintain the TE properties to modulate the DC pro- and anti-inflammatory cytokine production induced by LPS. In addition, TE or F<10 kDa treatment decreased the ability of immature DC to stimulate the allogeneic responses and induced a novo allogeneic CD4+CD25+Foxp3+ T cells. In contrast, treatment of DC with T/L or F<10 kDa plus LPS (F<10/L) induced a regulatory IL-27 dependent mechanism that diminished the proliferative and Th1 and Th17 allogeneic responses. Finally, we showed that a Kunitz type molecule (Fh-KTM), present in F<10 kDa, was responsible for suppressing pro-inflammatory cytokine production in LPS-activated DC, by printing tolerogenic features on DC that impaired their ability to induce inflammatory responses. These results suggest a modulatory role for this protein, which may be involved in the immune evasion mechanisms of the parasite.

## Introduction

The DC are the main antigen-presenting cells and are essential for initiating an effective adaptive response against diverse pathogens [1]. Unlike bacteria, helminth products do not mature DC in a conventional way, having been shown to possess the ability to suppress TLR-induced DC maturation [2] [3].

*F. hepatica* is a trematode parasite that causes disease in various ruminants, and is also an emergent disease in humans [4]. Different antigenic preparations from this parasite, such as tegumental or excretory-secretory products (ESP), are all capable of down-modulating TLR-induced DC activation, thus biasing the immune response toward an anti-inflammatory T regulatory/Th2 phenotype [5]–[7]. Among the excreted-secreted products of this parasite, cathepsin L1 cysteine protease (FhCL1) is one of the predominant secreted proteins, which has been shown to be involved in the down-regulation of LPS-induced macrophage maturation by TLR3 degradation, leading to a reduced TRIF-dependent MyD88-independent macrophage activation signaling pathway [8]. However, we have demonstrated that ESP and a somatic extract impair the DC activation induced by TLR ligands that involve both adaptor molecules (TRIF and MyD88) as well as non-TLR signaling [6], [7], suggesting that other molecules distinct from FhCL1 but present in *F hepatica* antigens modulate DC maturation. Considering that the parasite has to migrate through the host tissues and confront the inflammatory response induced by signals from both the parasite and exogenous environmental, it is reasonable to assume that distinct molecules, secreted or expressed by the parasite in its tegument, may participate during its migration in the prevention of an appropriate activation of DC, resulting in an anti-inflammatory control.

In this work, we have demonstrated the capacity of *Fh* total extract (TE) and a proteic fraction lower than 10 kDa (F<10 kDa) of TE to endow TLR-maturated DC with tolerogenic properties that promote T cell tolerance through an IL-27 dependent mechanism. It was also shown that both TE and F<10 kDa-treated DC were able to induce de novo Foxp3 T reg cells. Finally, we demonstrated that *F. hepatica* Kunitz serine protease inhibitor, a protein present in TE and in excretory-secretory products, as well as in the tegument of the parasite, is capable of printing regulatory features on TLR-induced activated DC, thereby impairing their ability to induce inflammatory responses.

## Results

### TE inhibits the LPS induced maturation and immunostimulatory capacity of murine and human DC

**Figure 1:**
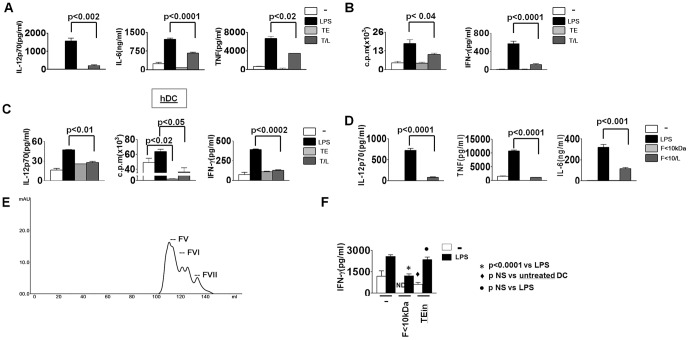
Proteins lower than 10 kDa from TE modulate mouse and human DC maturation and their allostimulatory capacity. (A) DC from BALB/c mice were incubated with medium, LPS (1 µg/ml), TE (80 µg/ml) or TE plus LPS (T/L) for 18 h and cytokine concentration was evaluated in the supernatants by ELISA. (B) DC from BALB/c mice treated as described in A, were co-cultured for 5 days with C57BL/6 splenocytes at a 1∶10 ratio. Allogeneic responses were determined by the proliferative 3[H]-thymidine incorporation assay and IFN-γ production by ELISA. (C) hDC from healthy donors treated with medium, LPS (100 ng/ml), TE (80 µg/ml) or T/L for 24 h were cultured with allogeneic PBMC at 1∶10 DC:PBMC cell ratio and the proliferative and IFN-γ response were tested. (D) IL-12p70, TNF and IL-6 were detected by ELISA in the supernatant of DC treated with medium or F<10 kDa (20 µg/ml) in the presence or absence of LPS for 18 h. (E) Elution profile of proteins lower than 10 kDa from TE, obtained by the ultrafiltration membrane with a cutoff lower than 10 kDa (F<10 kDa). (F) DC from BALB/c mice were treated with medium, F<10 kDa (20 µg/ml) or TE incomplete, without F<10 kDa (TEin, 80 µg/ml), and OVA peptide (0.5 µg/ml) in the presence or absence of LPS (1 µg/ml), before being cultured with splenocytes from DO11.10 TCR-transgenic mice, and IFN-γ production was evaluated after 5 days. Data show the mean of at least four wells ± SD, and are representative at least of two experiments with similar results, with p representing a significant difference in the Student's t-test.

In agreement with our previous studies [6], [7], antigens from *F. hepatica* negatively modulated the TLR-induced maturation of murine DC from BALB/c by reducing IL-12, IL-6, TNF production (Fig. 1A). Since the maturation status of DC is closely related to the type of adaptive response generated, it is feasible to hypothesize that LPS maturated DC in the presence of TE have less ability to initiate inflammatory adaptive responses than fully matured DC. Thus, we evaluated the capacity of these cells to initiate allogeneic responses. Although TE treatment did not modify the allogeneic stimulatory ability of immature DC, the T/L-treated DC did indeed reveal less capability than fully matured LPS-DC to induce a proliferative response or IFN-γ secretion against allogeneic cells (Fig. 1B), with IL-4 production being undetectable for all treatments (data not shown). In addition, as it had been established that TE treated DC had a reduced ability to initiate inflammatory responses to allo-antigens in culture using murine cells, we now wanted to extend these findings to human DC derived from PBMC (hDC). Therefore, hDC were treated in a similar way to murine DC and then cultured with human allogenic PBMC. In contrast with, LPS-treated hDC, TE plus LPS-treated hDC showed an attenuated capacity to secrete IL-12p70 and stimulate the proliferation and IFN-γ production of allogeneic PBMC, while TE-DC only induced a minor proliferative response compared to immature DC (Fig. 1 C). These data show that TE was able to prevent LPS induced DC activation, thereby initiating a defective adaptive inflammatory response.

### *F. hepatica* low molecular weight proteins are responsible for TE suppressor activity

In order to characterize the components present in TE able to modulate LPS-induced DC maturation, this complex mixture of proteins was separated into seven fractions by molecular exclusion chromatography using a Superdex G200 column (S1 Figure A). Then, the ability of each fraction to modulate the DC activation induced by LPS-maturated DC was tested by TNF production. The lowest molecular weight fraction, FVII (fraction seven), was found to be the only one able to significantly reduce the TNF production in LPS-treated DC (S2 Figure B), with DC treatment with a fraction mixture from I to V being unable to significantly reduce the levels of this cytokine induced by LPS (S1 Figure C). The fraction FVI was not included due to its proximity with FVII in the FPLC elution profile. Since FVII contained TE proteins lower than 10 kDa (data not shown), these were obtained by ultrafiltration (F<10 kDa). This latter fraction was capable of inhibiting the IL-12p70, TNF and IL-6 production induced by LPS in DC (F<10/L) (Fig. 1D), as had been demonstrated for TE, although F<10 kDa also contained proteins belonging to the FV and FVI peaks (Fig. 1E).

In a similar way to T/L-DC, F<10/L-DC revealed a decreased ability of immature and LPS-matured hDC to induce allogeneic responses, as was observed in the proliferation assays, regardless of the DC: PBMC cell ratio (S1 Figure D). Moreover, the treatment of DC with F<10 kDa also reduced the ability of immature or LPS-maturated DC to induce OVA specific IFN-γ production (Fig. 1 F). In contrast, proteins contained in TE, which were higher than 10 kDa (TEin), did not modify the IFN-γ response induced by untreated DC or LPS-DC (Fig. 1F), thus confirming the suppressor nature of F<10 kDa. In summary, proteins lower than 10 kDa were shown to be responsible for TE modulation of the DC ability to initiate inflammatory responses.

### TE treatment induces two different regulatory DC

**Figure 2:**
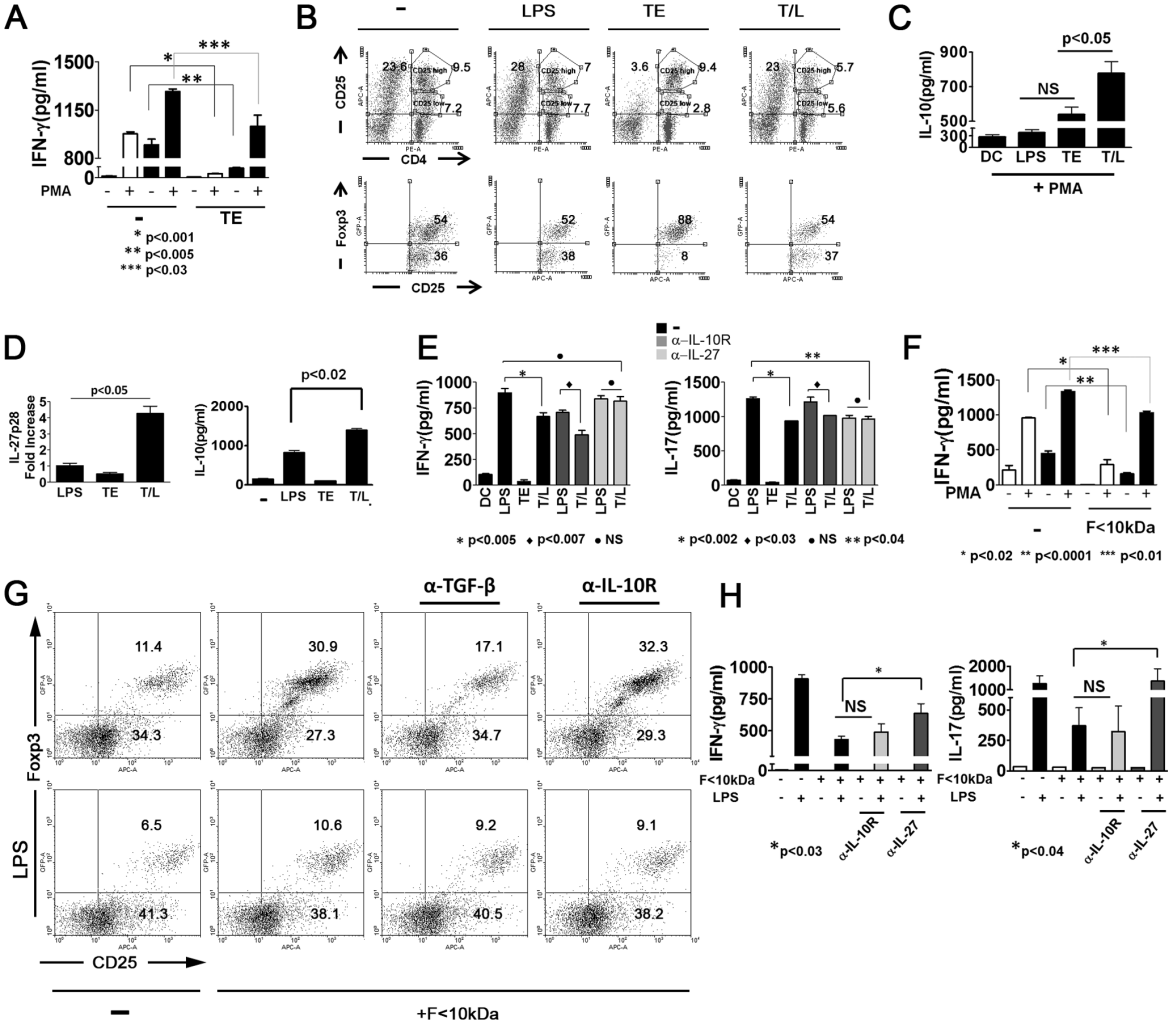
Reduced capacity of TE and F<10 KDa to prime allogeneic responses *in vitro*. (A) DC from BALB/c mice treated with medium, LPS (1 µg/ml), TE (80 µg/ml) or TE plus LPS for 18 h were co-cultured for 5 days with C57BL/6splenocytes at a 1∶10 ratio in the presence or absence of PMA. Allogeneic response was determined by IFN-γ production by ELISA. (B) DC from BALB/c mice, treated as described in A, without PMA, were cultured with a sorted EGFP negative cell population from splenocytes of Foxp3EGFP mice for 5 days. Dot plots show the CD4 vs CD25 profile of culture cells (upper panel) and the Foxp3 vs CD25 on the CD25 high gated cell population (lower panel). The numbers represent the percentage of Foxp3 positive and negative cells gated on the CD25 high cell population. (C) IL-10 was measured in the supernatant of allogeneic cultures as described in A in the presence of PMA for the last 18 h of culture, p<0.006 versus T/L-DC (ANOVA Test). (D) IL-27 mRNA was measured in DC treated with medium, LPS (1 µg/ml), TE (80 µg/ml) or TE plus LPS for 6 h by RT-PCR or IL-10 was detected in culture supernatant by ELISA after 18 h of culture, p<0.001 versus T/L-DC (ANOVA Test). (E). IFN-γ or IL-17 were detected in the supernatant of DC from BALB/c mice treated with medium, LPS, TE or TE plus LPS for 18 h, and cultured with splenocytes from C57BL/6 at a 1∶10 ratio for 5 days in the presence or absence of anti IL-10 R, anti-IL-27 or the control isotype Abs. Data are representative of three experiments, with p representing a significant difference in the Student's t-test. (F) IFN-γ was measured by ELISA in culture supernatants of splenocytes (C57BL/6) and DC (BALB/c mice), previously pulsed for 18 h with medium, or F<10 kDa (20 µg/ml) in the presence or absence of LPS (1 µg/ml) for 5 days. On day 4, the cells were stimulated with PMA for the last 18 h. (G) DC from BALB/c mice treated with medium or F<10 kDa (20 µg/ml) for 18 h were cultured with a sorted EGFP negative cell population from splenocytes of Foxp3EGFP in the presence or absence of anti- TGF β or anti IL-10R for 5 days. Dot plots show the Foxp3 *vs* CD25 profile of splenocytes gated on the CD4 population. (H) IFN-γ and IL-17 were detected in the supernatant of cultures as described in F, in the presence or absence of anti IL-27 or anti IL-10R blocking Abs. Data are representative of 2 experiments with similar results. The bar graphs shown one experiment representative of three with the mean ± SD. p represents a significant difference in the Student's t-test.

Since DC treated only with TE were unable to induce any detectable changes in the allogeneic response, compared to those induced by immature cells, we decided to repeat the allogeneic cultures in the absence or presence of a potent inflammatory stimulus such as PMA. As described in fig. 1B, immature or TE-treated DC had similar abilities to induce allogeneic response (Fig. 2A). Remarkably, the addition of PMA in the last 18 h of the co-culture of splenocytes with allogeneic TE-treated DC did not increase IFN-γ production as occurred with immature DC (Fig. 2 A). Although allogeneic splenocytes cultured with T/L-treated DC increased IFN-γ levels due to the addition of PMA, its production was significantly lower compared with the amount detected in cultures with LPS-treated DC in the presence of PMA (Fig. 2 A). This may indicate that the treatment with TE of both immature and LPS-matured DC printed on these cells a strong regulatory capacity to generate mechanisms which inhibited inflammatory allogeneic responses, even in the presence of a mitogen such as PMA.

In order to study whether both immature or LPS matured DC treated with TE were able to induce regulatory T cells, differentially treated DC were co-cultured with a Foxp3 negative cell population sorted from allogeneic splenocytes derived from Foxp3^EGFP^ C57BL/6 transgenic mice. The analysis of CD4^+^CD25^+^cells revealed two markedly different populations with respect to the CD25 expression percentage (Fig. 2 B), with Foxp3 expression occurring only in the CD25^+high^ population (data not shown). Remarkably, TE-treated DC induced a lower percentage of CD4^+^CD25^+low^ and CD4^-^CD25^+^ cell populations than the other treatments, while the percentage of the CD4^+^CD25^+high^ population was similar to that observed in the co-culture with immature DC (Fig. 2 B). Although immature, LPS- and T/L-treated DC were able to induce Foxp3 expression in allogeneic cultures (Fig. 2 B), TE-treated DC induced the highest percentage of positive cells for this regulatory transcription factor among all the treatments in the CD25^+^ high population (Fig. 2 B).

Since immature, LPS- or T/L-treated DC had similar abilities to induce CD4^+^CD25^+^Foxp3^+^ cells (Fig. 2 B), the T/L treatment should therefore promote additional mechanisms that are different from T reg induction in order to suppress the allogeneic responses. Related to this, we detected an increased IL-10 production in splenocytes cultured with allogeneic T/L-treated DC (Fig. 2C). Since IL-10-secreting CD4+ Tr1 cells are induced through IL-27 and IL-10 APC secretion, it prompted us to investigate whether these cytokines produced by DC, could be involved in the inhibition of Th1 allogeneic responses [9]. DC were treated with medium, LPS, TE or T/L, and the expression of IL-27p28 mRNA and IL-10 secretion were evaluated. The T/L treatment induced an up-regulation in the IL-27p28 subunit in DC compared with the rest of the treatments and revealed the highest IL-10 production (Fig. 2 D). In spite of both IL-27 and IL-10 being anti- inflammatory cytokines, only the blockage of IL-27 reverted the suppression of IFN-γ and IL-17 production induced by T/L-DC in allogeneic responses, with similar levels occurring as those induced by LPS treated DC (Fig. 2 E).

Finally, in order to determine whether TE-treated DC could modulate an antigen specific immune response, we cultured DC co-pulsed with TE and OVA peptide in the presence or absence of LPS, together with splenocytes from DO11.10 TCR-transgenic mice. T/L-treated DC revealed a lower capacity than LPS-treated DC to induce proliferative or IFN-γ responses of T cells to OVA peptide (S2 Figure A). Additionally, TE-treated DC promoted an increase in IL-4 production in co-cultures with CD4+ T cells (S2 Figure A and B), whereas T/L-treated DC induced an increase in IL-10 production in CD4+OVA-specific T cells (S2 Figure C). Similar to that observed in allogeneic cultures, TE-treated DC induced the greatest increase in the expression of Foxp3CD25+ T regulatory cells in an OVA specific T cell culture and also the highest CD25 expression in CD4+ cells (S2 Figure C).

These data show that TE is able to print a different regulatory hallmark to DC, depending on the absence or presence of an inflammatory stimulus such as LPS, thereby exerting control over the inflammatory T cell responses through possibly the induction of Foxp3+ regulatory cells or the secretion of IL-27.

### Antigens lower than 10 kDa from TE are responsible for two different regulatory DC inductions

To determine whether the treatment with F<10 kDa was able to induce two different types of regulatory DC, depending on the presence or absence of LPS, in a similar way to that found for TE, we performed allogeneic cultures with PMA. As in the case of TE, DC treated with F<10 KDa were able to maintain low levels of allogeneic IFN-γ production, even in presence of PMA, while immature DC were not capable of controlling this response (Fig. 2 F). As was observed with TE, the treatment with F<10 kDa, but not with F<10/L, enabled DC to induce the highest CD4+CD25+Foxp3+ regulatory T cell percentage in an allogeneic culture (Fig. 2 G), with this increased expression of Foxp3 being dependent on TGF-β but not on IL-10 (Fig. 2 G). Furthermore, the inhibition of the IFN-γ and IL-17 allogeneic responses induced by F<10/L-DC was IL-27 but not IL-10 dependent (Fig. 2 H). Nevertheless, the addition of blocking antibodies for TGF-β or IL-10R to the co-cultures did not modify the Foxp3 positive cell percentages induced by F<10/L-DC.

In summary, proteins lowers than 10 kDa were responsible for the TE ability to modulate immature or LPS-activated DC, thus conferring on these cells tolerogenic properties that can reduce the inflammatory adaptive responses.

### *F. hepatica* derived Kunitz-type protease inhibitor is an immunomodulatory protein

**Figure 3:**
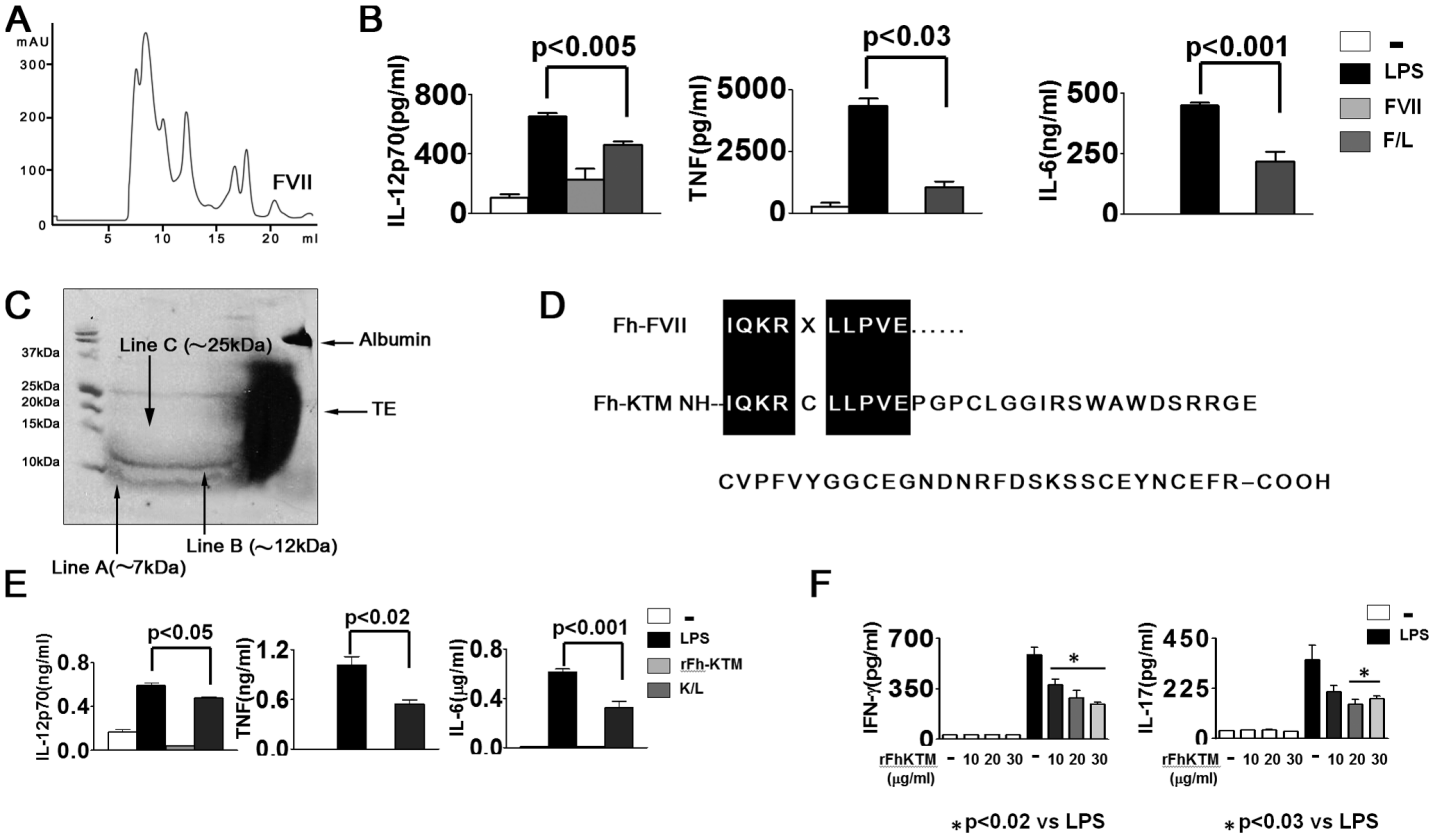
Kunitz type protease inhibitor present in FVII modulates TLR-induced DC activation (A). Elution profile of TE fractionated by Superdex G75. (B) IL-12p70, TNF and IL-6 production were detected by ELISA in the supernatants of DC cultured with medium, LPS (1 µg/mL), FVII (10 µg/ml) or FVII plus LPS (F/L) for 18 h. (C) FVII was resolved in 18% SDS-PAGE gel and stained with amido black (D) Comparison of the N-terminal sequences obtained for FVII and *F. hepatica* KTM. (E) DC were incubated with medium or rFh-KTM (20 µg/ml) in the presence or absence of LPS (1 µg/ml), and after 18 h the cytokine production was evaluated in the supernatants by ELISA. (F) IFN-γ and IL-17 were detected in the supernatant of DC from BALB/c mice treated with medium, LPS, rFh-KTM (10, 20 and 30 µg/ml) or rFh-KTM plus LPS for 18 h, and cultured with C57BL/6 splenocytes at a 1∶10 ratio for 5 days. Data are means ±SD of at least five wells and are representative results from two or three experiments. p represents a significant difference in the Student's t-test.

In order to isolate FVII and characterize its components, a superdex G75 column was utilized as it allows a better resolution for low molecular weight proteins. (Fig. 3 A). The FVII fraction was found to inhibit the IL-12p70, TNF and IL-6 production by LPS-treated DC (Fig. 3 B), and when FVII was resolved on reducing SDS-PAGE (18%), two bands of low molecular weight of about 7 and 12 kDa (lines A and B) were evident by amido black staining (Fig. 3 C), with the amino terminal also revealing a single sequence. Database searches determined that the N-terminal had a sequence identity (90%) to the Kunitz serine protease inhibitor from *F. hepatica* (Fh-KTM) (Fig. 3 D). Furthermore, a 25 kDa additional band (line C) with a low intensity was also observed (Fig. 3 C), but no sequence could be obtained due to the low amount of protein present. It is, however, possible that during the lyophilization process, monomeric aggregation of the protein occurred, thereby giving rise to the 12 and 25 bands.

Trying to confirm the involvement of Fh-KTM in the induction of suppressive properties on DC, the recombinant was assayed in DC cultures (rFh-KTM). Although rFh-KTM did not modify the pro-inflammatory cytokine secretion of immature DC, it was capable of reducing to inhibit, as in the case of F<10 kDa, the LPS-induced DC secretion of IL-12p70, TNF and IL-6 (Fig. 3 E). In an attempt to corroborate that rFh-KTM was capable of reducing the ability of DC to activate the adaptive inflammatory response, DC treated with rFh-KTM at different doses plus LPS (K/L-DC) were cultured with allogeneic splenocytes. Similar to that which occurred with T/L- and F<10/L-DC, the K/L-DC were less able than LPS-DC to induce IFN-γ or IL-17 allogeneic responses for all rFh-KTM doses tested (Fig 3 F). These results indicate that this protein of *F. hepatica*, which has Kunitz activity (data not shown), exerts a negative regulation on DC maturation induced by LPS in a similar way to that observed with TE, by being able to generate defective activated DC with a low production of pro-inflammatory cytokines and a poor capacity to initiate adaptive inflammatory responses. Therefore, our data support the idea of an immunomodulatory role for this protein and also of its possible involvement in immunoevasion mechanisms.

## Discussion

In this study we described the ability of a total extract from the helminth trematode *F. hepatica* to confer tolerogenic properties on human and mouse DC. Our results showed that in the presence of TE, DC remained immature, were resistant to LPS-induced maturation and decreased the allostimulatory capacity of LPS-treated DC. More importantly, we established that a proteic fraction lower than 10 kDa (F<10 kDa) from TE is responsible for the modulatory effect on DC.

Helminth parasites have acquired not only efficient mechanisms for infection and tissue invasion, but have also developed an immunomodulatory capacity allowing long-term residence in different guests. [10]. To survive in the host, *F. hepatica*, releases during its migration products from both the gut and tegument, which have been shown to be extensively involved in the modulation of immune responses [11]. Although the capacity of excretory-secretory products or tegumental antigens from *F. hepatica* to down-modulate DC activation has been previously reported [6] [12], few purified proteins that demonstrated an immunomodulatory activity on these cells in *F. hepatica* have been described.

Among the secreted proteins, cathepsin L (CL1) and the glutathione transferase (GST) have shown the ability to modulate DC maturation [13]. However, while both these proteins were capable of inducing the production of the inflammatory cytokines IL-12p40 and IL-6 in immature DC, they revealed an inhibitory effect only on the production of IL-23 in LPS-induced DC maturation [13]. Moreover, GST having been demonstrated to be part of the adult somatic extract, this protein is mostly expressed in the excretory-secretory products (ESP) [14]. In a similar way, CL1 has been described to be a major protein in the ESP from the adult parasite but also requires an acidification process to be active [15]. For all these reasons we think that although CL1 and GST could be present in TE, they would not be responsible for the observed modulatory effect on DC. Although both these proteins have shown a modulatory effect on DC maturation, neither has demonstrated a suppressive activity on the production of IL-12p70, TNF or IL-6 in TLR-induced mature APC. In contrast, a helminth defence molecule secreted by *F. hepatica* (Fh-HDM1) was able to down modulate IL-1β and TNF production by peritoneal macrophages in response to LPS binding directly to LPS, thereby reducing its interaction with both LPS-binding protein (LBP) and the surface of macrophages [16]. According to its molecular weight, Fh-HDM1 may be in the range F<10 kDa. However, we observed an inhibitory effect of TE and F<10 kDa not only on TLR-4 signaling but also TLR-2, -3 and -9 induced-DC maturation, as well as on non-TLR activators (data not shown), suggesting that a mechanism different from LPS binding or competition for the receptor was taking place.

To clarify the mechanism by which TE mediates its suppressive effect, we performed experiments that showed that in a similar way to ESP, TE also induced tolerogenic DC, which generated the novo CD4CD25Foxp3 regulatory cells [6]. The increased percentage of these cells in cultures of TE-DC with allogeneic splenocytes, could explained the fact that even in the presence of PMA, we did not observe an increase in the IFNγ production in contrast to immature DC. Moreover, this DC modulation was maintained only for the F<10 kDa fraction from TE. In addition, both TE- and F<10 kDa-DC induced a minimal percentage of CD4^+^CD25^int^ and CD4^−^CD25^+^ effector cells in allogeneic cultures, which indicates that these DC had tolerogenic properties absent in immature DC, which did not initiate regulatory mechanisms to control PMA response.

In the other hand, depending on whether an inflammatory stimulus was present, TE and F<10 kDa were able to induce two distinct regulatory DC. Thus, when LPS was added to TE or F<10 kDa-treated DC, these cells began to secrete IL-10 and IL-27, which have been widely described as regulatory cytokines [9]. However, only IL-27 blockage was able to restore the LPS induced-IFN-γ and IL-17 levels by allogeneic splenocytes. These data suggest that it was not only the activation status of DC that prevented these adaptive inflammatory responses, but also the presence of regulatory mechanisms, which were initiated by these antigens in order to control inflammation efficiently. In this study, we have described an essential IL-27 dependent mechanism that is exerted by *F. hepatica* antigens to suppress the immune system. Moreover, the fact that these regulatory pathways were IL-10 independent, could have been due to our experimental settings involving only the induction phase of adaptive response, while the release of IL-10 by T cells serves as a broad inhibitor on the activation of accessory cells and decreases the release of pro-inflammatory cytokines, resulting in reduced T cell activation and an ongoing inflammatory response [9]. Thus, DC exposed simultaneously to *F. hepatica* Ags and endogenous ligands (which activate TLR signaling), may then prevent the appropriate activation of these cells, thus resulting in an anti-inflammatory control during parasite migration.

Here, we have allocated an immune-regulatory function to a Kunitz type molecule described by Bozas as a trypsin inhibitor [17]. This molecule was able, in a similar way to TE and F<10 kDa, to decrease LPS-induced DC activation and DC ability to initiate Th1 and Th17 responses.

Kunitz protease inhibitors are a ubiquitous family of proteins, found in many organisms, including animals, plants and microbes [18]. At least three families of Kunitz inhibitors have been identified in vertebrates, which selectively inhibit serine protease activity and are involved in various anti-inflammatory processes [18]. Interestingly, protease inhibitors possess intrinsic properties that contribute to the termination of the inflammatory process, including modulation of cytokine expression, signal transduction and tissue remodeling [19]. It is increasingly apparent that the proteinase inhibitors of parasite origin can also modulate the host immune response against the parasite. In particular, for example cystatins from the helminth parasite *Onchocerca volvulus* induce anti-inflammatory responses, such as up-regulation of IL-10 or down-regulation of the expression of CD86 and HLA-DR on human monocytes [20] . Data published by Muiño [21] revealed that Fh-KTM binds to Cathepsin L, and proposed a possible immunoregulatory function for this protein. Additionally, as Dowling et al. [13] demonstrated that cathepsin L signals through TLR4, it may be hypothesized that Fh-KTM is stabilizing or promoting this union in some way, or alternatively Fh-KTM together with Cathepsin L could be acting simultaneously on TLR signaling to induce regulatory innate cells such as DC or macrophages. Additionally, inhibition of Fh-KTM might prevent DC activation by endogenous PRR ligands (products of necrotic cells or products from extracellular matrix disruption during parasite migration through the liver).

The restraint of Th1 and Th17 inflammatory responses by *F hepatica* has been reported in mice infected with the parasite, which exerts bystander suppression of immune responses to autoantigens and attenuates the clinical signs of experimental autoimmune encephalomyelitis [22]. In agreement with our findings, in the present study we have shown that Fh-KTM generate a DC phenotype able to impair Th1 and Th17 inflammatory responses, thus highlighting the role played by different molecules secreted by the parasite in order to reduce a hostile environment. Although, the precise mechanism by which this inhibition occurs is still not clear, our data show that IL-27 may be a key mediator in modulating the host immune response. In agreement with our results, the suppression of Th1 and Th17 responses and attenuation of experimental autoimmune encephalomyelitis by *F. hepatica* was maintained in IL-10(-/-) mice, but was reversed by neutralization of TGF-beta in vivo [22]. We also showed the novo Foxp3 T cell induction to be promoted by TE- or F<10 kDa-DC, which was TGF-beta dependent. Both, IL-27 and Foxp3, may occur in an infection scenario induced by Fh-KTM together with other molecules with redundant suppressor functions such as GST, CL1 or HDM.

Future experimental approaches using recombinant Fh-KTM may contribute to a better understanding of the mechanisms used by this molecule, which is abundantly expressed in the tegument, gut, ESP and parasite vomitus to modulate the innate and adaptive immune response. Thus, this protein might emerge as a new therapeutic target for the control of exacerbated inflammatory responses.

## Materials and Methods

### Human cells

Peripheral blood mononuclear cells (PBMC) were obtained from healthy consenting donors, according to protocols approved by the institutional Review Board of Foundation for the advancement of Medicine and by The Private Hospital of Córdoba. Institutional review board waived the need for consent.

### Animals and TE preparation

Six- to eight-week-old inbred female BALB/c and C57BL/6 mice were purchased from the Faculty of Veterinary Sciences, National University of Litoral (UNL, Argentina), and Foxp3^EGFP^ mice (all of the C57BL/6 background) and DO11.10 OVA transgenic mice, were obtained from Jackson Laboratories (Bar Harbor, ME). The Institutional Experimentation Animal Committee (authorization n°15-01-44195) approved animal handling and experimental procedures. TE of *F. hepatica* was obtained from mature flukes of infected bovine livers from the slaughterhouse Bustos and Beltrán (Córdoba, Argentina), as previously described with some modifications [23]. Briefly, adult *F. hepatica* worms were homogenized by mechanical disruption in a tissue grinder, and the supernatant was obtained at 10000×g. To remove endotoxin contamination, a sample of TE was applied to a column containing detoxi-gel endotoxin removing gel (Pierce Biotechnology, Rockford USA). After endotoxin removal, the quantity of LPS present in TE was determined by using the Limulus amebocyte lysate test (Endosafe Times Charles River, Laboratories Wilmington, Delaware), whose level was found to be similar to those of the background and complete RPMI 1640 medium. The protein concentration was quantified by using a Bio-Rad Protein assay (CA, USA) in the sample eluted from the column. For the *in vitro* assays, the TE was diluted between 20 or 40 times, in order to obtained a final concentration of 80 µg/ml in the wells.

### TE fractioning and recombinant Fh-KTM obtention

The TE was fractionated by size exclusion fast protein liquid chromatography (FPLC) on an ÄKTAFPLC Purifier UPC 10 (GE Healthcare Life Science, Sweden) system with a Superdex 200 HR 10/30 or Superdex 75 column (Amersham Bioscience, USA) in the PBS buffer (pH 7.4) and the fractions (25–30) were then collected.

To obtain F<10 kDa, TE 20 ml (6 mg/ml) was passed through an ultrafiltration membrane (GE Health Care, UK) with a molecular weight cut-off (MWCO) of 10 kDa in a microcentrifuge. The recombinant of Fh-KTM (rFh-KTM) was ordered by Shanghai Apeptide Co. Ltd. (Shangai, China), according to a sequence described by Bozas et. al. [17]. Endotoxin contamination was removed in both antigens by a detoxi-gel endotoxin removing gel (Pierce Biotechnology, Rockford USA), with the endotoxin level found being similar to those of the background and complete RPMI 1640 medium.

### Mice DC generation and stimulation

The DC were generated as previously described, with slight modifications [24]. Mice were sacrificed by cervical dislocation, and bone marrow was collected from femurs of mice, and the cells were cultured with 7.5% of supernatant from GM-CSF, which produced J558 cells (20 ng/ml final concentration in the plate) in 8 days. After this time period, harvested cells comprised 85% DC (Class II^+^, CD11c^+^). Expression of CD11c was quantified by flow cytometry using FITC- or PE-conjugated Ab purchased from BD Biosciences. To activate the DC, 4×10^5^ cells were treated with TE (80 µg/ml), F<10 kDa (20 µg/ml), FVII (10 µg/ml) or rFh-KTM (10, 20 or 30 µg/ml) in the presence or absence of 1 µg/ml of LPS extracted from *E. coli* (serotype 055:B5; Sigma-Aldrich. Then, after 16 h of culture, the supernatants were collected for cytokine measurement. Cell viability was assessed by using an annexin V-FITC apoptosis detection kit (BD, Biosciences, San Diego, CA) with the dead-cell dye 7-AAD (Santa Cruz Biotechnology; San Diego, CA). The viability of DC after all treatments was about 75–90%.

### Cytokine measurement

Cytokines were detected in culture supernatants using capture ELISA. IFN-γ (Biosource, CA USA), IL-10, IL-12p70, IL-17 and IL-4 (eBioscience, USA), TNF and IL-6, (BD Pharmingen, USA) were used as paired mAb in combination with recombinant cytokine standards. Assays were performed according to the manufacturer's guidelines.

### Allogeneic mixed lymphocyte reaction

DC from BALB/c mice unstimulated or previously treated with TE, F<10 kDa or rFh-KTM (10. 20 and 30 µg/ml) in the presence or absence of LPS were cultured for 18 h, before being irradiated with 30 Gy. Collected cells were cultured with C57BL/6 wild type in the presence or absence of 10 µg/ml of the anti-IL10R (BD Pharmingen, USA) or anti IL-27 blocking Abs (R&D Systems, USA) for 5 days or transgenic Foxp3^EGFP^ splenocytes (2×10^5^ cells/well) for 5 days at a 10∶1 ratio (DC:splenocyte). In some cultures, PMA (Sigma Aldrich, USA) (1 µg/ml) was added to the wells of DC plus allogeneic splenocytes for the last 24 h of culture. For proliferation studies, after 96 h of culture, wells were pulsed with 1 µCi 3H-thymidine (Amersham, Life Science, Buckinghamshire, UK), and the cultures harvested 18 h later using an automated cell harvester. 3H-thymidine incorporation into the DNA of cells was measured using a beta liquid scintillation counter. In some cultures, the FoxP3^EGFP-^ splenocytes, with a purity higher than 98% from Foxp3^EGFP^ mice were sorted by using flow cytometer FACSAria™ IIu (BD San Jose, CA). Then, the cells were cultured with allogeneic DC from BALB/c mice unstimulated or previously treated with TE, LPS, TE plus LPS, F<10 kDa, or with F<10 KDa plus LPS, in the presence or absence of antibody anti-TGF-β (R&D Systems, USA), anti-IL10 (BD Pharmingen, USA) for 5 days. The cells were then analyzed using the antibodies anti-CD4 (eBiosciences, USA), anti-CD25 (eBiosciences, USA) and FoxP3EGFP+ on the lymphocyte region by FACS canto II (BD, San Jose, CA). Data were analyzed using Flowing software 2.5.0. (Turku Centre for Biotechnology University of Turku, Finland).

### qPCR

Total cellular RNA was prepared using Trizol from unstimulated or TE-, LPS- or TE plus LPS treated DC for 18 h, and cDNA synthesis was performed as previously described [25]. Briefly, RNA was isolated from 5×10^6^ DC using TRIZOL reagent (Invitrogen) as indicated by the suppliers. Two micrograms of total RNA were reverse transcribed to cDNA according to conventional methods in a total volume of 25 µl with the reaction taking place for 1 h at 42°C and being terminated by boiling for 5 min. All reagents were obtained from Fermentas Life Science (Tecnolab). The cDNA produced was amplified by PCR using specific primers for IL-27. As an endogens control, the GAPDH primer was used, with each primer pair being tested to achieve the appropriate conditions of amplification.

The qPCR analysis was performed using an ABI Prism 7500 detection system (Applied Biosystems, Foster City, CA) and SYBR green chemistry. Reactions were carried out in triplicate using, for each 15- µl reaction, 5 µl of cDNA template (equivalent to 250 ng of total RNA), 0.3 µm forward and reverse primers for IL27p28 (forward sequence CCCTGAGCCTTCAAGAGCTGCG; reverse sequence CAGCAAAGCTGTGGACATAGCCCTGAAC) and Fast-Plus EvaGreen qPCR master mix (Biotium, CA, USA). To quantify any changes in gene expression, the 2−ΔΔCt method was used to calculate the relative changes, which were normalized against the housekeeping gene GADPH used as an internal control. For each pair of primers, a dissociation plot resulted in a single peak, indicating that the single cDNA product was amplified. All primers were purchased from Genbiotech (Buenos Aires, Argentina).

### N-terminal amino acid sequence of FVII proteins

To analyze the N-terminal amino acid sequence, FVII was resolved by 18% SDS-PAGE under reducing conditions and transferred onto polyvinylidene difluoride (PVDF) membranes (Millipore, MA USA), as described by Matsudaira [26]. Sequence analysis of FVII proteins was performed in the 'Laboratorio Nacional de Peptidos y Proteínas (LANAIS-PRO, Buenos Aires, Argentina), using an Applied Biosystems Model 477A pulsed-liquid sequencer. The amino acid sequence was screened for homologies with known sequences in the GenBank, EMBL and Swiss Prot database, using the Fasta algorithm.

### Isolation of CD14+ monocytes and differentiation of human monocyte derived DC (hDC)

Peripheral blood mononuclear cells (PBMC) were isolated from buffy coat by Ficoll-Hypaque gradient (GE Healthcare Bio-Sciences) from anonymous blood samples, and CD14+ monocytes were purified using CD14+ mAb-conjugated magnetic beads (MACS MicroBeads; Miltenyi Biotech), according to the manufacturer's protocol [27] . Immature hDC were generated by culturing CD14+ monocytes in RPMI 1640 medium containing 10% FBS (Invitrogen), 800 U/ml GM-CSF and 500 U/ml IL-4 (BD Biosciences) for 5 days, which resulted in more than 90% CD11c+ cells. The medium was replaced on day 3. For maturation, hDC were stimulated with TE (80 µg/ml) and F<10 kDa (20 µg/ml) in the presence or absence of LPS (100 ng/ml) for 18 h. The supernatants were collected and IL-12p70 was detected by ELISA.

### Mixed Leukocyte Reaction

The allostimulatory capacity of the hDC was tested in a mixed leukocyte reaction (MLR). Allogeneic peripheral blood mononuclear cells (PBMC) were co-cultured with differently matured hDC in a 96-well tissue culture microplate, and the proliferative response was assessed at various hDC:PBMC cell ratios after 5 days by measuring the thymidine incorporation (1 µCi/ml [methyl-3H]thymidine; specific activity, 50 Ci/mmol; New England Nuclear). Supernatants from hDC: PBMC cell co-cultures (ratio 1∶10) were harvested at 24 hours and analyzed for IFN-γ release by ELISA (eBioscience).

### OVA-specific response

DC from BALB/c mice were cultured with medium, F<10 kDa (20 µg/ml) or TE incomplete (without F<10 kDa, TEin, 80 µg/ml), and OVA peptide (0.5 µg/ml) in the presence or absence of LPS (1 µg/ml), and then were cultured with splenocytes from DO11.10 TCR-transgenic mice and IFN-γ production was evaluated after 5 days. Cells were stained with FITC–conjugated anti-CD4 mAb, or APC-conjugated anti-CD25 mAb (BD-Pharmingen). For intracellular IL-10, IL-4 and Foxp3 staining, the cells were cultured for 5 hours with PMA (10 ng/ml) and ionomycin (1 mg/ml; Sigma-Aldrich) and brefeldin A (10 mg/ml; Sigma-Aldrich) was added for the last 4 hours of cell culture. The cells were stained for PE- or APC-conjugated anti-Foxp3 (e-Bioscience) and PE-conjugated anti IL-10 antibody or anti IL-4 (BD-Pharmingen) using Foxp3 Fixation/Permeabilization. Concentrate and diluent and permeabilization buffer (e-Bioscience) and analyzed by FACS.

### Statistical Analysis

The measurement of cytokines in the DC supernatant was performed for three wells per culture, with 3 to 5 cultures being included in each group and the data expressed as means ± standard deviation. The Student's *t*-test was used for statistical comparisons. In some experiments data were analyzed with one way ANOVA Dunnett's multiple comparison test. For both tests p-values <0.05 were considered significant.

## Supporting Information

S1 Figure The lowest molecular weight fraction from TE modulates mouse DC maturation and the ability of human DC to induce allogeneic responses (A) Elution profile of TE fractionated by Superdex G200 into seven fractions. (B) TNF was detected in the supernatant of DC cultured with LPS (1 µg/ml) in the presence of each fraction (I–VII) (10 µg/ml) for 18 h. (C) or TNF was measured by ELISA in the supernatants of DC, cultured with medium, FVII (10 µg/ml), or a mixture of fractions of one to five (FI–V) (30 µg/ml) in the presence or absence of LPS (1 µg/ml) for 18 h. (D) Immature (left) or LPS-matured hDC (right) were co-treated with F<10 kDa (20 µg/ml) for 18 h and then were cultured with allogenic PBMC at different cells ratios and proliferative response was evaluated. Data are means ±SD of triplicate wells and are representative results from three experiments.
(TIF)

S2 Figure TE and F<10 kDa inhibit the capacity of LPS-maturated DC to induce the OVA-specific T cell response (A) DC from BALB/c mice were treated with medium or TE (80 µg/ml) and OVA peptide (0.5 µg/ml) in the presence or absence of LPS (1 µg/ml), together with splenocytes from DO11.10 TCR-transgenic mice. The OVA specific T cell response was evaluated by [3H] thymidine incorporation, IFN-γ and IL-4 production were detected by ELISA in cultures after 5 days. The values shown are the means of triplicates ± SD. The experiment was carried out twice and one representative experiment is given. (B) IL-10 and IL-4 production was measured by FACS in CD4+ T cells in cultures as described in A by intracellular cell staining. Gray histograms show isotype control stained cell population (C) Cells from cultures as described in A were stained to detect CD4, CD25 and Foxp3 expression, and CD4+Foxp3 (left panel) CD4+CD25 (right panel) are shown. Gray histograms show the cultures of immature OVA-pulsed DC plus splenocytes from DO11.10 TCR-transgenic mice.
(TIF)
