# Supplementary figures and images for: Fasciola hepatica Kunitz Type Molecule Decreases Dendritic Cell Activation and Their Ability to Induce Inflammatory Responses
Source: PLoS One. 2014 Dec 8;9(12):e114505. doi: 10.1371/journal.pone.0114505 (PMC4259355; doi:10.1371/journal.pone.0114505)

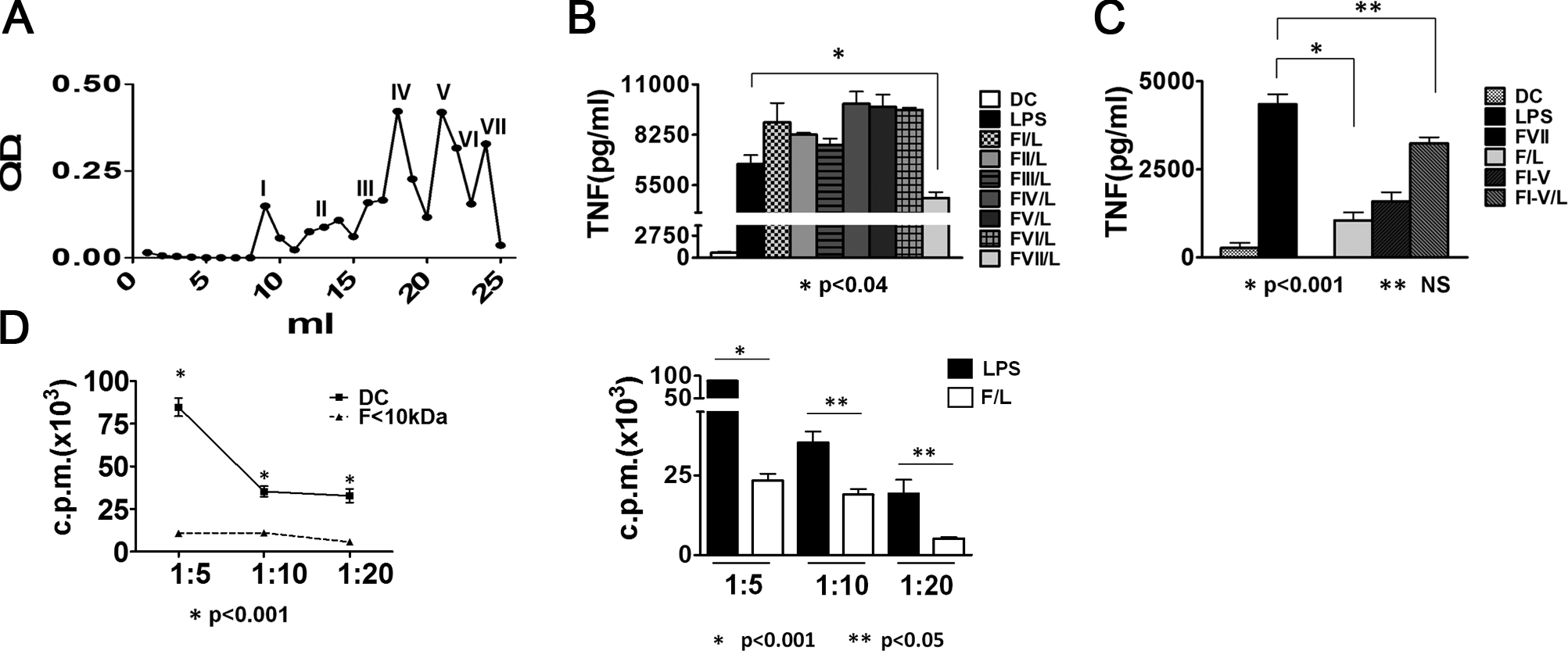

Supplement: S1 Figure — The lowest molecular weight fraction from TE modulates mouse DC maturation and the ability of human DC to induce allogeneic responses (A) Elution profile of TE fractionated by Superdex G200 into seven fractions. (B) TNF was detected in the supernatant of DC cultured with LPS (1 µg/ml) in the presence of each fraction (I–VII) (10 µg/ml) for 18 h. (C) or TNF was measured by ELISA in the supernatants of DC, cultured with medium, FVII (10 µg/ml), or a mixture of fractions of one to five (FI–V) (30 µg/ml) in the presence or absence of LPS (1 µg/ml) for 18 h. (D) Immature (left) or LPS-matured hDC (right) were co-treated with F<10 kDa (20 µg/ml) for 18 h and then were cultured with allogenic PBMC at different cells ratios and proliferative response was evaluated. Data are means ±SD of triplicate wells and are representative results from three experiments. (TIF) [file pone.0114505.s001.tif]

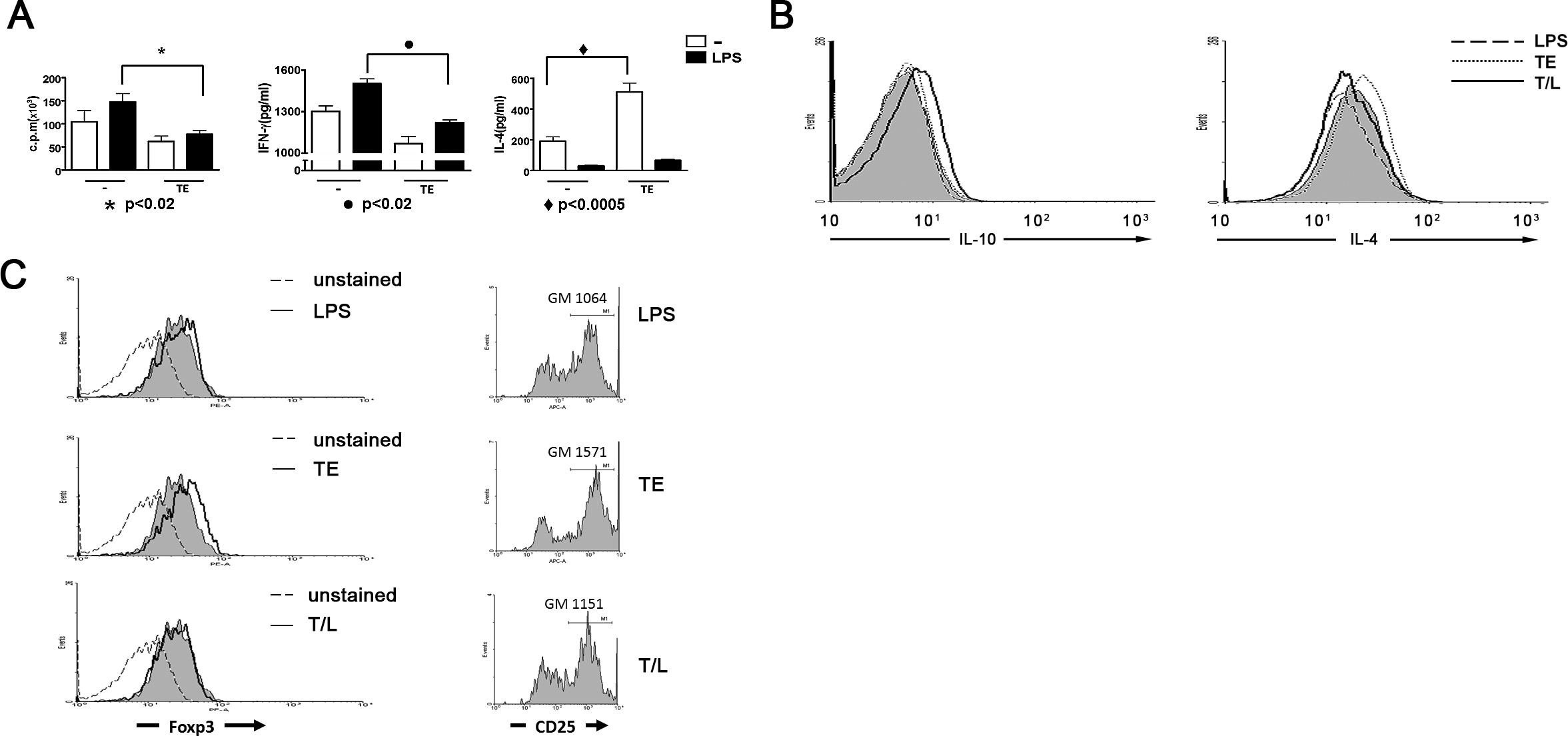

Supplement: S2 Figure — TE and F<10 kDa inhibit the capacity of LPS-maturated DC to induce the OVA-specific T cell response (A) DC from BALB/c mice were treated with medium or TE (80 µg/ml) and OVA peptide (0.5 µg/ml) in the presence or absence of LPS (1 µg/ml), together with splenocytes from DO11.10 TCR-transgenic mice. The OVA specific T cell response was evaluated by [3H] thymidine incorporation, IFN-γ and IL-4 production were detected by ELISA in cultures after 5 days. The values shown are the means of triplicates ± SD. The experiment was carried out twice and one representative experiment is given. (B) IL-10 and IL-4 production was measured by FACS in CD4+ T cells in cultures as described in A by intracellular cell staining. Gray histograms show isotype control stained cell population (C) Cells from cultures as described in A were stained to detect CD4, CD25 and Foxp3 expression, and CD4+Foxp3 (left panel) CD4+CD25 (right panel) are shown. Gray histograms show the cultures of immature OVA-pulsed DC plus splenocytes from DO11.10 TCR-transgenic mice. (TIF) [file pone.0114505.s002.tif]
